# CT-Visible Convexity Subarachnoid Hemorrhage Predicts Early Recurrence of Lobar Hemorrhage

**DOI:** 10.3389/fneur.2022.843851

**Published:** 2022-03-23

**Authors:** Qiong Yang, Xiangzhu Zeng, Zhou Yu, Xiaolu Liu, Lu Tang, Gaoqi Zhang, Danyang Tian, Nan Li, Dongsheng Fan

**Affiliations:** ^1^Department of Neurology, Peking University Third Hospital, Beijing, China; ^2^Department of Radiology, Peking University Third Hospital, Beijing, China; ^3^Research Center of Clinical Epidemiology, Peking University Third Hospital, Beijing, China; ^4^Beijing Key Laboratory of Biomarker and Translational Research in Neurodegenerative Diseases, Beijing, China

**Keywords:** intracerebral hemorrhage, cerebral amyloid angiopathy, convexity subarachnoid hemorrhage, recurrence, prognosis

## Abstract

**Background and Purpose:**

Convexity subarachnoid hemorrhage (cSAH) may predict an increased recurrence risk in cerebral amyloid angiopathy (CAA)-related intracerebral hemorrhage (ICH) survivors. We aimed to investigate whether cSAH detected on CT was related to early recurrence in patients with ICH related to CAA.

**Methods:**

We analyzed data from consecutive lobar ICH patients diagnosed as probable or possible CAA according to the Boston criteria using the method of cohort study. Demographic and clinical data, ICH recurrence at discharge and within 90 days were collected. The association between cSAH detected on CT and early recurrent ICH was analyzed using multivariable logistic regression.

**Results:**

A total of 197 cases (74 [66–80] years) were included. cSAH was observed on the baseline CT of 91 patients (46.2%). A total of 5.1% (10/197) and 9.5% (17/179) of patients experienced ICH recurrence within 2 weeks and 90 days, respectively. The presence of cSAH was related to recurrence within 2 weeks (OR = 5.705, 95%CI 1.070–30.412, *P* = 0.041) after adjusting for hypertension, previous symptomatic ICH and anticoagulant use. The presence of cSAH was related to recurrence within 90 days (OR 5.473, 95%CI 1.425–21.028, *P* = 0.013) after adjusting for hypertension, previous symptomatic ICH and intraventricular hemorrhage. The similar results were obtained in other models using different methods to select adjusting variables.

**Conclusion:**

In patients with lobar ICH related to CAA, 5.1% and 9.5% of them experienced ICH recurrence within 2 weeks and 90 days, respectively. CT-visible cSAH was detected in 46.2% of patients and indicates an increased risk for early recurrent ICH.

## Introduction

Cerebral amyloid angiopathy (CAA) is a cerebral small vessel disease characterized by the deposition of amyloid-β in small cortical and leptomeningeal vessels (1). It is, in elderly individuals, a leading cause of lobar intracerebral hemorrhage (ICH), which is a devastating condition associated with substantial disability, mortality and dementia (1, 2). The risk of recurrent ICH is still high, despite preventive management including strict blood pressure control and bleeding risk-based antithrombotic therapy (1–3), and accurate recurrence risk stratification may help to improve the clinical management of these patients.

Convex subarachnoid hemorrhage (cSAH) occurs within the subarachnoid space of cortical sulci. In addition to the isolated cSAH which is in the absence of parenchymal blood, cSAH is sometimes observed with lobar ICH (3, 4) and is recognized as an important marker for CAA according to the Edinburgh diagnostic criteria (5). Cortical superficial siderosis (cSS), chronic hemosiderin staining of the pial surface visible on susceptibility-weighted imaging (SWI), is considered a late consequence of cSAH and a marker of rupture of leptomeningeal vessel as a result of advanced CAA (6, 7).

Previous studies have shown that the presence and progression of cSS can predict the future risk of ICH in CAA patients (8–14). Further studies showed that cSAH on magnetic resonance imaging (MRI) (whether isolated cSAH or cSAH accompanied by acute ICH) is a predictor of future ICH (15, 16). However, the application of MRI in the acute phase is limited in clinical practice. A recent study showed that cSAH detected on non-contrast CT can predict the recurrence risk after discharge in patients with CAA-related ICH (17).

The above studies evaluated cSAH in CAA-related ICH survivors at discharge (16, 17), so there may be survivor bias if the patient had in-hospital recurrence or death in the early stage after ICH. On the contrary, studies focus on early recurrence may be helpful to identify predictors and promote early intervention. Previous data showed a skewed distribution of recurrence patterns concentrating in the early stage after ICH (18). The recurrence risk was higher after lobar ICH than after non-lobar ICH (19), and some CAA patients appeared to experience early recurrence weeks after lobar ICH (13, 20). As a marker of advanced CAA, cSAH may also be a marker of disease activity and predict early recurrence after the initial ICH. On this basis, we aimed to investigate whether cSAH detected on CT is related to early recurrence in patients with CAA-related ICH.

## Materials and Methods

### Study Setting

We analyzed the data of a prospective cohort of consecutive acute primary ICH patients from 19 hospitals in Beijing and its surrounding areas in northern China. Our inclusion criteria were as follows: (1) acute lobar hemorrhage; (2) diagnosed as probable or possible CAA according to the Boston criteria (21); and (3) available baseline brain CT scan. Patients were excluded if they had brain surgery before the baseline CT. In addition, lobar ICH patients with probable or possible CAA admitted to our center during the same period who were not included in the above multicenter cohort were enrolled according to the same inclusion and exclusion criteria.

This study was approved by the Ethics Committee of Peking University Third Hospital, and informed consent was obtained from the participants or their legal representatives.

### Data Collection

Demographic and baseline clinical data including vascular risk factors, pre-stroke medications, history of ICH, NIHSS score, treatment, and hospital length of stay were collected. APOE genotypes were determined from the patients' donated blood samples for genetic testing.

### Image Acquisition and Analysis

**Figure 1:**
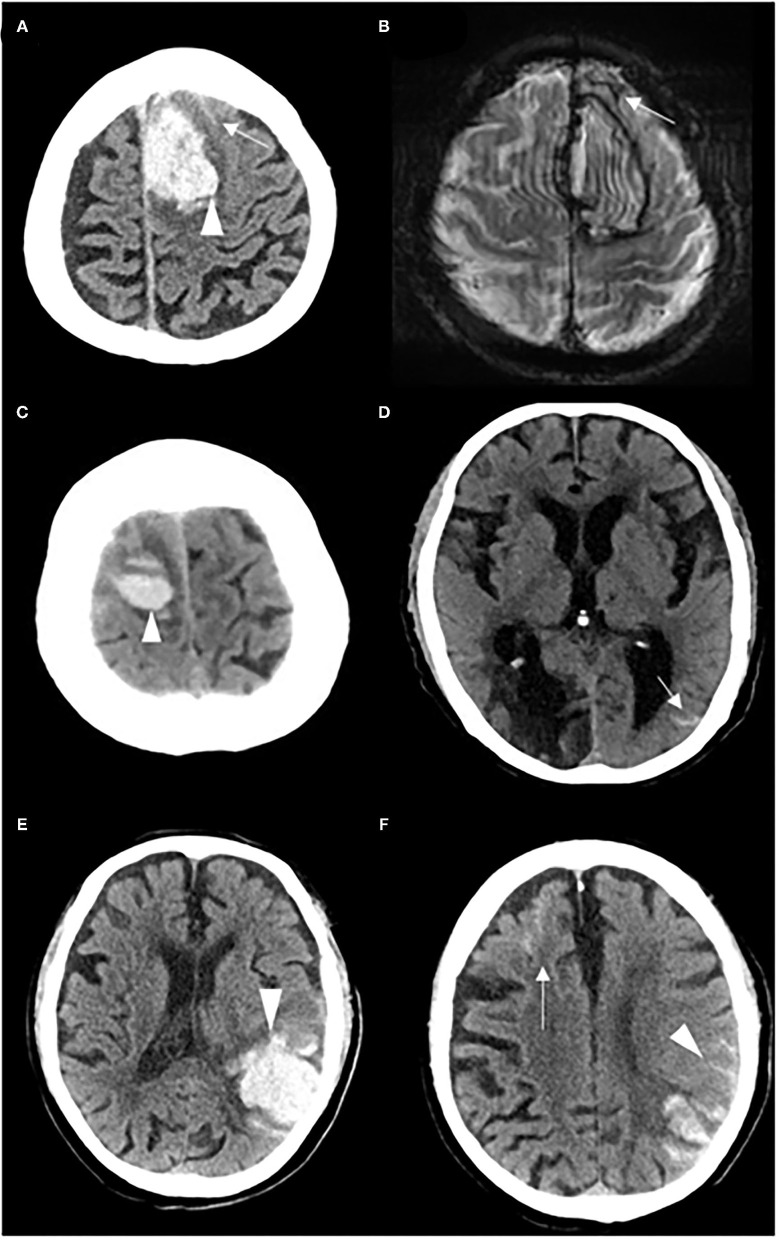
Representative examples of adjacent cSAH and remote cSAH. **(A)** A patient with adjacent cSAH: cSAH (white arrow) was observed within 1–2 sulci from the acute left frontal ICH (white head of arrow), and **(B)** cSS (white arrow) on the T2*-weighted gradient-recalled echo at 1 month after the index ICH. **(C,D)** A patient with remote cSAH: cSAH (white arrow) was detected on the left side, remote from the acute right frontal ICH (white head of arrow). **(E,F)** A patient with remote cSAH: cSAH (white arrow) was detected in the left frontal lobe, remote from the acute left temporal parietal ICH (white head of arrow), with adjacent cSAH (white head of arrow).

All CT scans were reviewed by radiologists who were blinded to the baseline and follow-up data to determine the location and volume of the ICH and the presence of intraventricular hemorrhage (IVH). cSAH was defined as a linear high-density signal in the subarachnoid space on CT that could be adjacent to or remote from the ICH (17). cSAH extent was classified as follows: (1) adjacent cSAH: bleeding was strictly confined within 1 or 2 sulci from the acute hematoma; and (2) remote cSAH: cSAH was >2 unaffected sulci away from the acute ICH, with or without adjacent cSAH (Figure 1) (16, 17).

The presence and extent of cSAH on CT were visually assessed by 2 investigators (Q.Y. and X.Z.) The interrater agreement was good for cSAH presence (kappa = 0.753), as well as for both adjacent cSAH (kappa = 0.720) and remote cSAH (kappa = 0.718). Discrepancies were settled by consensus after the investigators independently read all of the scans.

Brain MRIs were acquired on 3.0T scanners including bloodsensitive sequence (T2^*^ GRE or SWI) and fluid-attenuated inversion recovery (FLAIR). All MRIs were assessed by radiologists who were blinded to the baseline and follow-up data according to the Standards For Reporting Vascular Changes on Neuroimaging (STRIVE) (22). cSS was defined as curvilinear chronic blood products in the superficial layer of the cerebral cortex that showed linear hypointensities on T2^*^GRE/SWI images and with no corresponding hyperintense signal on T1-weighted or FLAIR images (16). The distribution of cSS was classified as focal (restricted to ≤ 3 sulci) or disseminated (≥4 sulci) (16, 23). Cerebral microbleeds (CMBs) were defined as small hypointense lesions on T2^*^ GRE/SWI images and the number and distribution were assessed (22). White matter hyperintensities (WMH) including periventricular hyperintensities and deep hyperintensities were assessed using the Fazekas rating scale (24). Enlarged perivascular spaces (EPVS) were assessed in the basal ganglia and the centrum semiovale with a 4-point visual rating scale (0 = no EPVS, 1 = <10 EPVS, 2 = 11–20 EPVS, 3 = 21–40 EPVS, and 4 = > 40 EPVS). The degree of EPVS was classified as high (score ≥ 3) or low (score ≤ 2) (22, 25).

### Follow-Up

Follow-up data were collected at discharge in the hospital and at 90 days through face-to-face or telephone interviews with the patients and their caregivers. Data including the presence of recurrent ICH and death after the index ICH were collected. Recurrent ICH was defined as a new ICH affecting a separate region in the brain. All reports of recurrent ICH were confirmed by CT scan, and the location of the recurrent ICH was recorded.

### Statistical Analysis

Categorical variables are presented as counts (%), and continuous variables are presented as medians with interquartile ranges (IQR, 1st quartile to 3rd quartile) due to the nature of the underlying distribution. The baseline characteristics, including demographic, clinical and imaging data, were compared between patients with and without cSAH and between those with and without recurrent ICH. The χ^2^ test or Fisher's exact test were used for categorical variables, and the Mann-Whitney U test was used for continuous variables, as appropriate. Factors associated with cSAH were identified using multivariable logistic regression. The distribution of the time of ICH recurrence was analyzed using system cluster analysis. Factors associated with ICH recurrence were identified using multivariable analysis including three kinds of models. In model 1A, variables with a *P*-value <0.1 in the univariate logistic regression were included, and in Model 2A, prespecified plausible predictors of recurrent ICH according to literature search and clinical inference (including age, previous ICH, previous use of antiplatelets and anticoagulants before ICH, and cSAH) were included for multivariable logistic regression. In Model 3A, the prespecified plausible predictors as well as variables with a *P*-value <0.1 in univariable regression were included, and backward (conditional) logistic regression were used, considering the number of outcomes was relatively small. Based on the above models, the association between the extent of cSAH (adjacent or remote ICH) and ICH recurrence was analyzed in Models 1B, 2B, and 3B for further analysis. Multicollinearity was measured using variance inflation factors, and predictors with variance inflation factors >5 were removed from the model. Statistical significance was defined as P <0.05. The statistical analyses were performed using SPSS (version 22.0).

## Results

### Patient Demographics

**Figure 2:**
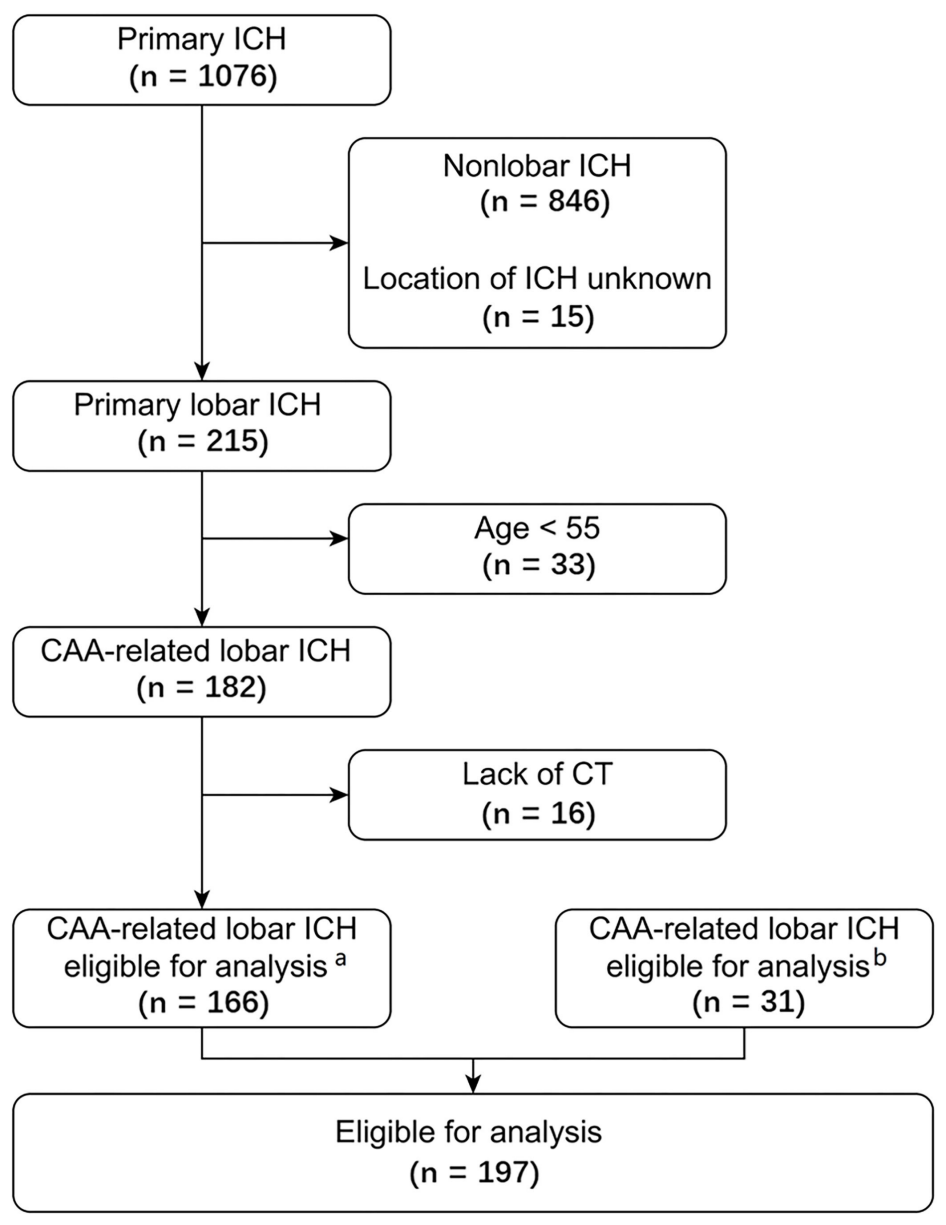
Flow chart of the patient selection process. ^a^Patients included in the multicenter cohort. ^b^Patients admitted to our center during the same period who were not included in the multicenter cohort. CAA, cerebral amyloid angiopathy; ICH, intracerebral hemorrhage.

Of 1,085 patients with primary ICH in the multicenter cohort, 179 lobar ICH patients fulfilled the Boston criteria for possible or probable CAA. Among them, 166 patients were eligible, and 13 were excluded because of unavailable CT scans. In addition, 31 lobar ICH patients with probable or possible CAA admitted to our hospital during the same period who were not included in the above multicenter cohort were eligible and enrolled. In summary, a total of 197 patients (aged 74 [66–80] years, 114 males [57.9%]) were included in the final analysis: 50 with probable CAA, and 147 with possible CAA according to the Boston criteria using CT scan. There were 17 patients (8.6%) died during hospitalization. One hundred and eighty five patients were achieved 90-day followed-up, among which 24 died (13.0%). A flow diagram of the patient selection process in the current analysis is presented in Figure 2.

### Prevalence of cSAH on CT and Associated Factors

cSAH was present in 91 of 197 patients (46.2%). Of the 91 patients with cSAH, 74 (81.3%) had adjacent cSAH, and 17 (18.7%) had remote cSAH.

The comparison of patients with and without cSAH is shown in Table 1. Compared to patients without cSAH, patients with cSAH were older (79 vs. 71, P < 0.001), had a larger baseline ICH volume (29.8 ml vs. 13.8 ml, P < 0.001), a higher incidence of IVH (37.4% vs. 17.9%, *P* = 0.002), and a higher NIHSS score (7 vs. 2, *P* = 0.001). In addition, more patients with cSAH received surgical treatment (20.9% vs. 10.4%, *P* = 0.041), and these patients had a longer hospital length of stay (22 vs. 17, *P* = 0.037).

**Table 1:** Baseline characteristics and comparison of patients with and without cSAH.

	**Whole ICH cohort**	**Patients with cSAH**	**Patients without cSAH**	***P*-value**
No. of patients		*n =* 91	*n =* 106	
**Demographics**				
Age, Median [IQR]	74 [66–80]	79 [71–82]	71 [62–78]	<0.001
Male, No. (%)	114(57.9)	50 (54.9)	64 (60.4)	0.441
**Clinical characteristics**				
Hypertension, No. (%)	112 (56.9)	46 (50.5)	66 (62.3)	0.098
Diabetes mellitus^a^, No. (%)	39 (20.5)	20 (22.5)	19 (18.8)	0.533
Hyperlipidemia^b^, No. (%)	23 (13.6)	8 (10.1)	15(16.7)	0.216
Previous symptomatic ICH, No. (%)	40 (20.3)	17 (18.7)	23(21.7)	0.600
Previous antiplatelet use^c^, No. (%)	33 (17.9)	14 (16.3)	19 (19.4)	0.583
Previous anticoagulant use^d^, No. (%)	2 (1.1)	1(1.2)	1(1.0)	1.000
NIHSS score^e^, Median [IQR]	3 [1–13]	7 [1–19]	2 [1–6]	0.001
Surgical treatment, No. (%)	30 (15.2)	19 (20.9)	11 (10.4)	0.041
Hospital length of stay^f^, Median [IQR]	19 [14–29]	22 [14–36]	17 [14–26]	0.037
ICH volume (mL), Median [IQR]	19.7 [7.3–42.2]	29.8 [12.2–59.6]	13.8 [4.0–29.2]	<0.001
Intraventricular hemorrhage presence, No. (%)	53 (26.9)	34 (37.4)	19 (17.9)	0.002
APOE ε2 (≥1 copy)^g^, *n* (%)	30 (24.2)	16 (27.6)	14 (21.2)	0.408
APOE ε4 (≥1 copy)^g^, *n* (%)	31 (25.0)	16 (27.6)	15 (22.7)	0.533

a *7 patients with missing data*.

b *28 patients with missing data*.

c *13patients with missing data*.

d *7 patients with missing data*.

e *30 patients with missing data*.

f *3 patients with missing data*.

g *124 patients consented to APOE genotype testing*.

*cSAH, convexity subarachnoid hemorrhage; ICH, intracerebral hemorrhage; IQR, interquartile range*.

Multivariate analysis showed that age (OR 1.094, 95% CI 1.050–1.140, P <0.001) and ICH volume (OR 1.018, 95% CI 1.002–1.035, *P* = 0.032) were independently associated with cSAH presence, while hypertension (OR 0.908, 95% CI 0.453–1.820, *P* = 0.786), IVH (OR 2.069, 95% CI 0.847–5.055, *P* = 0.111), and NIHSS score (OR 1.031, 95% CI 0.992–1.073, *P* = 0.123) were not.

### Time Distribution of Early Recurrent ICH

Within 90 days of onset, 17 patients had recurrent ICH, and all of them are lobar hemorrhage. Excluding one patient who developed recurrence after discharge (18 days after onset) with an uncertain recurrence time, the time of recurrence for the other 16 patients was classified into 3 groups by cluster analysis: 10 patients recurred within 2 weeks, 4 patients within 2 weeks to 2 months, and 2 patients within 2 to 3 months. The patients were still in the early stage of treatment and rehabilitation within 2 weeks after onset, and most of the recurrences (10/17 = 58.8%) occurred in this stage. Therefore, our study analyzed the factors related to recurrence within 2 weeks.

### cSAH and Risk of Recurrent ICH Within 2 Weeks

A total of 5.1% (10/197) of patients had ICH recurrence within 2 weeks. In univariate logistic analysis, previous symptomatic ICH (OR 4.343, 95% CI 1.192–15.823, *P* = 0.026), previous anticoagulant use (OR = 19.889, 95% CI 1.149–344.360, *P* = 0.040), presence of cSAH (OR = 5.012, 95% CI 1.036–24.239, *P* = 0.045), and presence of adjacent cSAH (OR = 4.179, 95% CI 1.046–16.697, *P* = 0.043) were associated with recurrence within 2 weeks (Table 2).

**Table 2:** Univariate regression analysis of factors for recurrent ICH within 2 weeks.

**Variables**	***P***	**OR**	**95% CI**
Age	0.544	1.023	0.950–1.102
Male	0.430	0.573	0.114–2.286
Hypertension	0.094	0.307	0.077–1.223
Diabetes mellitus^a^	0.966	0.966	0.197–4.743
Hyperlipidemia^b^	0.823	0.784	0.093–6.578
Previous symptomatic ICH	0.026	4.343	1.192–15.823
Previous antiplatelet use^c^	0.316	2.057	0.503–8.414
Previous anticoagulant use^d^	0.040	19.889	1.149–344.360
NIHSS score^e^	0.513	1.020	0.961–1.084
ICH volume	0.176	1.012	0.995–1.030
Presence of cSAH	0.045	5.012	1.036–24.239
Adjacent cSAH	0.043	4.179	1.046–16.697
Remote cSAH	0.874	1.187	0.141–9.978
Intraventricular hemorrhage presence	0.345	1.878	0.508–6.934

a *7 patients with missing data*.

b *28 patients with missing data*.

c *13 patients with missing data*.

d *7 patients with missing data*.

e *30 patients with missing data*.

*cSAH, convexity subarachnoid hemorrhage; ICH, intracerebral hemorrhage*.

In multivariate analysis (model 1A), cSAH presence (OR = 5.705, 95% CI 1.070–30.412, *P* = 0.041) was associated with recurrence within 2 weeks after adjusting for hypertension, previous symptomatic ICH and previous anticoagulant use (Table 3).

**Table 3:** Multivariate regression analysis of predictors of ICH recurrence within 2 weeks.

**Variables**	**P**	**OR**	**95%CI**	**Variables**	**P**	**OR**	**95% CI**
**Model 1A^a^**				**Model 1B^a^**			
Hypertension	0.161	0.348	0.080–1.523	Hypertension	0.174	0.356	0.080–1.578
Previous symptomatic ICH	0.009	6.721	1.601–28.217	Previous symptomatic ICH	0.012	6.458	1.520–27.443
Previous anticoagulant use	0.035	51.496	1.316–2015.073	Previous anticoagulant use	0.014	93.154	2.538–3418.992
cSAH presence	0.041	5.705	1.070–30.412	Adjacent cSAH	0.029	6.924	1.213–39.529
				Remote cSAH	0.711	1.874	0.068–51.744
**Model 2A^b^**				**Model 2B^b^**			
Age	0.371	1.044	0.950–1.148	Age	0.393	1.043	0.947–1.149
Previous symptomatic ICH	0.003	12.035	2.271–63.777	Previous symptomatic ICH	0.004	12.151	2.262–65.271
Previous antiplatelet use	0.050	5.839	1.003–33.972	Previous antiplatelet use	0.041	6.413	1.080–38.064
Previous anticoagulant use	0.007	144.703	3.840–5453.485	Previous anticoagulant use	0.004	278.839	6.357–12231.088
cSAH presence	0.029	6.846	1.216–38.554	Adjacent cSAH	0.019	8.974	1.432–56.234
				Remote cSAH	0.576	2.336	0.119–45.809
**Model 3A^c^**				**Model 3B^c^**			
Previous symptomatic ICH	0.004	9.988	2.088–47.772	Previous symptomatic ICH	0.005	10.504	2.061–53.539
Previous antiplatelet use	0.068	4.800	0.893–25.803	Previous antiplatelet use	0.035	6.767	1.148–39.881
Previous anticoagulant use	0.011	84.419	2.732–2608.185	Previous anticoagulant use	0.003	230.495	6.750–7870.852
cSAH presence	0.027	6.851	1.246–37.672	Hypertension	0.110	0.275	0.056–1.339
				Adjacent cSAH	0.032	6.356	1.168–34.588

a *Variables with a P-value < 0.1 in the univariate logistic analysis were included. Hypertension, previous symptomatic ICH, previous anticoagulant use and cSAH presence were included in model 1A. The first 3 variables, adjacent cSAH and remote cSAH were included in model 1B. Seven patients were excluded because of missing data*.

b *Prespecified plausible predictors of recurrent ICH were included. Age, previous symptomatic ICH, previous antiplatelet use, previous anticoagulant use, cSAH presence were included in model 2A. The first 4 variables, adjacent cSAH and remote cSAH were included in model 2B. 15 patients were excluded because of missing data*.

c *Prespecified plausible predictors as well as variables with a P-value < 0.1 in univariable regression were included using backward logistic regression. Age, hypertension, previous ICH, previous antiplatelet use and anticoagulant use, and cSAH were entered, among which 2 variables, including age and hypertension were eliminated in the final model as backward logistic regression in model 3A. Age, hypertension, previous ICH, previous antiplatelet use and anticoagulant use, adjacent and remote cSAH were entered, among which 2 variables, including age and remote cSAH were eliminated in the final model as backward logistic regression in model 3B. Fifteen patients were excluded because of missing data*.

*cSAH, convexity subarachnoid hemorrhage; ICH, intracerebral hemorrhage*.

In the prespecified multivariable analysis (model 2A), cSAH presence was associated with recurrence within 2 weeks (OR 6.846, 95% CI 1.216–38.554, *P* = 0.029) after adjusting for age, previous symptomatic ICH, and previous use of antiplatelets and anticoagulants (Table 3).

Age, hypertension, previous ICH, previous antiplatelet use and anticoagulant use, and cSAH were entered, among which 2 variables, including age and hypertension were eliminated in the final model as backward logistic regression in model 3A, and cSAH presence was associated with recurrence within 2 weeks (OR 6.851, 95% CI 1.246–37.672, *P* = 0.027) (Table 3).

Based on the above models, the association between the extent of cSAH and ICH recurrence was analyzed in Models 1B, 2B, and 3B which showed that adjacent cSAH was associated with recurrence within 2 weeks (OR = 6.924, 95% CI 1.213–39.529, *P* = 0.029; OR 8.974, 95% CI 1.432–56.234, *P* = 0.019; and OR 6.356, 95% CI 1.168–34.588, *P* = 0.032, respectively), while remote cSAH not (Table 3).

### cSAH and Risk of Recurrent ICH Within 90 Days

Follow-up data on recurrent ICH wasn't obtained for 18 patients (6 patients died, 12 patients were lost to follow-up), and 17 patients (9.5%) of the remaining 179 patients had recurrent ICH within 90 days.

In univariate analysis, hypertension (OR 0.301, 95% CI 0.101–0.896, *P* = 0.031), previous symptomatic ICH (OR 2.958, 95% CI 1.043–8.387, *P* = 0.041), cSAH (OR 6.290, 95% CI 1.740–22.741, *P* = 0.005), and adjacent cSAH (OR 6.152, 95% CI 1.916–19.752, *P* = 0.002) were associated with recurrence within 90 days (Table 4).

**Table 4:** Univariate regression analysis of factors for recurrent ICH within 90 days^a^.

**Variables**	***P***	**OR**	**95% CI**
Age	0.153	1.046	0.983–1.112
Male	0.988	0.993	0.360–2.739
Hypertension	0.031	0.301	0.101–0.896
Diabetes mellitus^b^	0.403	0.521	0.113–2.405
Hyperlipidemia^c^	0.350	0.371	0.047–2.964
Previous symptomatic ICH	0.041	2.958	1.043–8.387
Previous antiplatelet use^d^	0.965	1.030	0.276–3.843
Previous anticoagulant use^e^	0.112	9.812	0.585–164.479
NIHSS score^f^	0.202	1.032	0.983–1.083
ICH volume	0.193	1.010	0.995–1.026
cSAH presence	0.005	6.290	1.740–22.741
Adjacent cSAH	0.002	6.152	1.916–19.752
Remote cSAH	0.755	0.716	0.088–5.840
Intraventricular hemorrhage presence	0.055	2.711	0.980–7.497

a *18 patients were excluded because of missing data on ICH recurrence within 90 days*.

b *5 patients with missing data*.

c *24 patients with missing data*.

d *11 patients with missing data*.

e *4 patients with missing data*.

f *22 patients with missing data*.

*cSAH, convexity subarachnoid hemorrhage; ICH, intracerebral hemorrhage*.

In multivariate analysis (model 1A), cSAH presence was associated with recurrence within 90 days (OR 5.473, 95% CI 1.425–21.028, *P* = 0.013) after adjusting for hypertension, previous symptomatic ICH and IVH (Table 5).

**Table 5:** Multivariate regression analysis of predictors of ICH recurrence within 90 days.

**Variables**	***P***	**OR**	**95% CI**	**Variables**	***P***	**OR**	**95% CI**
**Model 1A^a^**				**Model 1B^a^**			
Hypertension	0.077	0.358	0.115–1.116	Hypertension	0.069	0.344	0.109–1.084
Previous symptomatic ICH	0.049	3.087	1.003–9.504	Previous symptomatic ICH	0.042	3.290	1.041–10.397
IVH presence	0.441	1.545	0.511–4.677	IVH presence	0.368	1.676	0.545–5.155
cSAH presence	0.013	5.473	1.425–21.028	Adjacent cSAH	0.007	6.562	1.680–25.631
				Remote cSAH	0.722	1.554	0.137–17.608
**Model 2A^b^**				**Model 2B^b^**			
Age	0.287	1.041	0.968–1.121	Age	0.306	1.040	0.965–1.121
Previous symptomatic ICH	0.008	5.129	1.542–17.056	Previous symptomatic ICH	0.007	5.509	1.611–18.839
Previous antiplatelet use	0.387	1.906	0.442–8.222	Previous antiplatelet use	0.270	2.335	0.518–10.536
Previous anticoagulants use	0.037	32.126	1.242–830.844	Previous anticoagulant use	0.011	80.008	2.748–2329.227
cSAH presence	0.007	6.573	1.666–25.935	Adjacent cSAH	0.003	8.732	2.053–37.131
				Remote cSAH	0.793	1.448	0.091–22.985
**Model 3A^c^**				**Model 3B^c^**			
Previous symptomatic ICH	0.018	3.989	1.266–12.576	Previous symptomatic ICH	0.016	4.347	1.321–14.299
Hypertension	0.056	0.326	0.103–1.027	Previous anticoagulant use	0.014	53.011	2.252–1248.103
cSAH presence	0.006	6.388	1.695–24.074	Hypertension	0.063	0.329	0.102–1.061
				Adjacent cSAH	0.003	7.868	2.055–30.123

a *Variables with a P-value < 0.1 in the univariate logistic analysis were included. Hypertension, Previous symptomatic ICH, IVH presence, cSAH presence were included in model 1A. The first 3 variables, adjacent cSAH and remote cSAH were included in model 1B. Eighteen patients were excluded because of missing follow-up data on recurrence*.

b *Prespecified plausible predictors of recurrent ICH were included. Age, previous symptomatic ICH, previous antiplatelet use, previous anticoagulant use, cSAH presence were included in model 2A. The first 4 variables, adjacent cSAH and remote cSAH were included in model 2B. 30 patients were excluded because of missing data*.

c *Prespecified plausible predictors as well as variables with a P-value < 0.1 in univariable regression were included using backward logistic regression. Age, hypertension, previous ICH, previous antiplatelet use and anticoagulant use, IVH, and cSAH were entered, among which 4 variables, including age, previous antiplatelet and anticoagulants use, and IVH were eliminated in the final model as backward logistic regression in model 3A. Age, previous antiplatelet use and anticoagulant use, IVH, hypertension, previous ICH, adjacent and remote cSAH were entered, among which 4 variables, including age, antiplatelet use, IVH and remote cSAH were eliminated in the final model as backward logistic regression in model 3B. Thirty patients were excluded because of missing data*.

*cSAH, convexity subarachnoid hemorrhage; ICH, intracerebral hemorrhage; IVH, intraventricular hemorrhage*.

In prespecified multivariable analysis (model 2A), cSAH presence was associated with recurrence within 90 days (OR 6.573, 95% CI 1.666–25.935, *P* = 0.007) after adjusting for age, previous symptomatic ICH, and previous use of antiplatelets and anticoagulants (Table 5).

In model 3A age, hypertension, previous ICH, previous antiplatelet use and anticoagulant use, IVH, and cSAH were entered, among which 4 variables, including age, previous antiplatelet and anticoagulants use, and IVH were eliminated in the final model as backward logistic regression, and cSAH presence was significantly associated with recurrence within 90 days (OR 6.388, 95% CI 1.695–24.074, *P* = 0.006) (Table 5).

Further analysis in Models 1B, 2B, and 3B showed that adjacent cSAH was associated with recurrence within 90 days (OR 6.562, 95% CI 1.680–25.631, *P* = 0.007; OR 8.732, 95% CI 2.053–37.131, *P* = 0.003; OR 7.868, 95% CI 2.055–30.123, *P* = 0.003, respectively) (Table 5).

### Supplemental Analyses of Patients With Probable CAA Diagnosed Based on CT Scan

In addition to the above analyses of both patients with probable and possible CAA, supplemental analyses of 50 patients with probable CAA diagnosed based on CT scan were performed. cSAH was present in 25 patients (50%). The comparison of patients with and without cSAH is shown in Supplementary Table 2.

A total of 16.0% (8/50) of patients had ICH recurrence within 2 weeks. In univariate logistic analysis, presence of cSAH (OR 3.632, 95% CI 0.656–20.115, *P* = 0.149), adjacent cSAH (OR 3.718, 95% CI 0.771–17.938, *P* = 0.102) and remote cSAH (OR 0.857, 95%CI 0.089–8.268, *P* = 0.894) were not associated with recurrence within 2 weeks (Supplementary Table 3).

A total of 25.0% (12/48) of patients had ICH recurrence within 90 days. In univariate analysis, cSAH (OR 7.857, 95%CI 1.495–41.302, *P* = 0.015) and adjacent cSAH (OR 9.000, 95% CI 1.991–40.791, *P* = 0.004) were associated with recurrence within 90 days (Supplementary Table 4). In multivariate analysis, cSAH was associated with recurrence within 90 days in Models 1A, 2A, and 3A (OR 6.791, 95%CI 1.254–36.777, *P* = 0.026; OR 6.553, 95% CI 1.165–36.870, *P* = 0.033; and OR 8.077, 95% CI 1.523–42.834, *P* = 0.014, respectively), so was adjacent cSAH in Models 1B, 2B and 3B (OR 9.434, 95% CI 1.627–53.647, *P* = 0.012; OR 9.814, 95% CI 1.606–59.959, *P* = 0.013; and OR 12.500, 95% CI 2.258–59.192, *P* = 0.004, respectively) (Supplementary Table 5).

### Supplemental Analyses of Patients With Available MRI

There were 81 patients with available MRI including T2^*^ GRE or SWI. Supplemental analyses of them were performed. cSAH detected on CT was present in 32 patients (39.5%). The comparison of patients with and without cSAH is shown in Supplementary Table 6.

A total of 6.2% (5/81) of patients had ICH recurrence within 2 weeks. In univariate logistic analysis, presence of cSAH (OR 2.431, 95% CI 0.383–15.431, *P* = 0.346), adjacent cSAH (OR 2.885, 95% CI 0.453–18.362, *P* = 0.262) and remote cSAH (*P* = 0.999) were not associated with recurrence within 2 weeks, so were other MRI markers including the presence of cSS (OR 5.806, 95% CI 0.619–54.463, *P* =0.124), the presence and count of CMB (OR 1.091, 95% CI 0.172–6.912, *P* = 0.926; and OR 1.015, 95% CI 0.843–1.091, *P* = 0.697, respectively), and Fazekas WMH score (OR 1.336, 95%CI 0.713–2.504, *P* = 0.366) (Supplementary Table 7).

A total of 11.7% (9/77) of patients had ICH recurrence within 90 days. In univariate logistic analysis, the presence of adjacent cSAH (OR 4.476, 95%CI 1.021–19.630, *P* = 0.047) and cSS (OR 5.315, 95%CI 1.026–27.532, *P* = 0.047) were associated with recurrence within 90 days, while the presence of cSAH (OR 3.667, 95%CI 0.841–15.986, *P* = 0.084) were not (Supplementary Table 8).

In multivariate analysis including presence of cSS and presence of cSAH or adjacent cSAH, none of them was associated with recurrence within 90 days (Supplementary Table 9).

## Discussion

In our cohort study of patients with lobar ICH related to CAA, cSAH was detected on CT in 46.2% of patients and was an important prognostic marker indicating an increased risk for the early recurrence of ICH.

The prevalence of cSAH based on CT in our study was slightly higher than that in previous studies (40.5%) (17) that included survivors, and the study design requiring MRI may lead to a selection bias toward less severe cases. The prevalence of cSAH based on CT in the above 2 studies was lower than that based on MRI (63.6%) (16). However, considering that CT is the most commonly used imaging method for patients with acute ICH and that the feasibility of MRI imaging is limited when scanners are unavailable and the procedure is unable to be tolerated or contraindicated, CT-based SAH evaluations may be more suitable for the early assessment of the risk of ICH recurrence.

Our study showed that CT-visible cSAH was an independent predictor of early recurrence of ICH in patients with lobar ICH related to CAA. The presence of cSS, whether focal or disseminated, can predict the risk of future ICH (8–14). cSS and cSAH are likely 2 imaging markers of the same pathophysiologic process of subarachnoid hemorrhage in CAA (17). Isolated cSAH was a predictor of an increased risk for future ICH in CAA patients (15, 26). Two recent studies showed that cSAH accompanied by acute ICH on MRI or CT was a predictor of long-term recurrence of ICH in survivors with CAA-related ICH (16, 17). The CT-based study showed that 20.0% of patients had recurrent ICH, and cSAH was an independent predictor of recurrent ICH (HR 2.64) (17). In addition to the above studies focusing on the long-term recurrence, a study showed that cSAH predicted the recurrent risk within 6 months, but the cSAH assessed in this study did not include cSAH adjacent to ICH (13). Our study supports that adjacent cSAH is a predictor of early recurrence, adding to the findings of previous studies that adjacent cSAH predicts long-term recurrence (16, 17).

Adjacent cSAH is often considered to result from the extension of lobar ICH into the subarachnoid space (3, 16). However, a few studies suggested that the occurrence of lobar ICH may begin with the rupture of superficial vessels in the leptomeninges which often have the heaviest burden of amyloid in CAA patients (27–29). Primary hemorrhage sometimes occurs in the subarachnoid space (27, 28) and major lobar hemorrhage may develop directly from or in extension of cSAH (29). Adjacent cSAH caused by the rupture of vessels in the leptomeninges may reflect vascular fragility and may be associated with a high recurrence risk.

However, our study did not show that remote cSAH was associated with ICH recurrence, which was the same as reported by Li et al. (17). The results regarding the association between remote cSAH and recurrence risk have been inconsistent. In an MRI-based study remote cSAH and adjacent cSAH showed similar associations with increased recurrence risk (16). One of the potential explanations would be due to the different imaging methods used to detect cSAH. The prevalence of remote cSAH based on MRI was 26.1%, which was higher than studies base on CT (11.9% in Li's study and 8.6% in ours) (16, 17). In addition, cSAH remote from the ICH may result from either a separate vascular rupture which might be associated with ICH recurrence, or the redistribution of blood in the subarachnoid space which is less likely to be the case (17). The relationship between remote cSAH and ICH recurrence as well as the potential mechanism remain uncertainty and need further investigations.

Previous studies on the pathology of CAA have suggested the mechanism of recurrence in patients with cSAH. cSS was associated with CAA changes in the leptomeningeal vessels, which usually have the heaviest amyloid-β burden (6, 7). cSS and cSAH may represent the different stages of subarachnoid hemorrhage in CAA caused by the rupture of leptomeningeal vessels as a result of advanced CAA (6, 7). The potential explanations included that the anatomic features of these vessels (such as vulnerability to loss of trophic factors because of fewer surrounding neuropils and less mechanical support because they are on the surface of the brain) may also make them vulnerable to rupture, which needs to be further tested (3).

The patients in our study had a high risk for early recurrence. In total, 9.5% patients developed recurrence within 90 days. Among them, 58.8% of patients experienced recurrence within 2 weeks. A study showed that the recurrence risk for primary ICH was concentrated in the early stage: the cumulative recurrence risk was 3.67% in 4 weeks, 5.86% at 3 months, and 8.86% at 1 year (18). Some CAA patients experience early recurrence after ICH, and these cases seem to be concentrated in the first 3–6 months (13, 20). During a mean follow-up of 28.1 months, 38.2% (21/55) of 55 recurrent lobar ICH occurred within 6 months (13). Therefore, more active observation of and prevention strategies for ICH recurrence (such as strict management of blood pressure and weighing the benefits and risks of antithrombotic drugs) in patients with acute lobar ICH is needed in clinical practice, and further studies are also needed to identify the predictors of early recurrence for inclusion as research targets for intervention therapy.

The patients had a high risk for early recurrence, which may be due to the following reasons: 1) different study populations: this study included patients died in the hospital, thus supplementing the data of previous studies that included survivors with that of patients with severe hemorrhage; and 2) ethnic differences: the APOE genotype is the most important genetic factor of CAA identified thus far, and its effects differ by race (30, 31). Studies have shown that APOE ε2 or ε4 allele carriers have an increased risk of incident and recurrent ICH (32). ε2 can promote CAA-related vasculopathy, which can lead to vessel rupture; thus, ε2 may be a stronger risk factor for CAA development than ε4 (33, 34). In our study, the proportion of ε2 carriers was 24.2% (27.6 and 21.2% in patients with and without SAH, respectively), which was higher than that in a previous study that reported a proportion of 18.5% (23.4% vs. 9.6% in patients with and without SAH, respectively), which may be a reason for the high recurrence in this cohort.

Our study had limitations. First, the sample size of our study was relatively small, the number of recurrent ICHs was limited, and some data were missing, including NIHSS score, hyperlipidemia, etc. For 9.1% (18/197) of the patients, the presence of recurrence could not be determined at the 90-day follow-up. Second, APOE allele profiles were available in only 62.9% (124/197) of the patients in our study; therefore, this potential predictor of ICH recurrence was not fully adjusted for in our multivariable analysis. Another limitation was that the diagnose of possible or probable CAA by Boston criteria in our study was based on CT scan because the MRI data only available in part of the patients. A proportion of patients of isolated lobar ICH are hypertensive-related but would be diagnosed as possible CAA using CT scan. Our supplemental analyses restricted in patients of probable CAA showed similar results that the cSAH and adjacent cSAH was related to ICH recurrence within 90 days, but not related to ICH recurrence within 2 weeks, for which one explanation might be relative lower number of recurrent events within 2 weeks. Our supplementary analysis restricted in patients with available MRI data did not show the correlation between cSAH and ICH recurrence in multivariate analysis, which might be due to the small number of cases and events. The validity of the diagnosis based on MRI is higher than CT, while it is sometimes not available in emergency setting, the reasons for which include unable to tolerate (for example, due to severe disease status), contraindication or MRI imaging not feasible in some hospitals. Therefore, it's reasonable to balance the validity and feasibility. MRI evaluation should be performed when available, while cSAH based on CT scan could be an alternative imaging marker to evaluate the prognosis when MRI is not available in the early stage after ICH. Studies with larger sample sizes are needed to validate our findings and to identify the underlying mechanisms in the future.

In summary, our study showed that in patients with lobar ICH related to CAA, 5.1 and 9.5% of them experienced ICH recurrence within 2 weeks and 90 days, respectively. cSAH is detected in 46.2% of patients on non-contrast CT and is an imaging marker associated with early ICH recurrence. This marker could be useful in stratifying early recurrence risk when only CT scans are available in the acute setting in clinical practice.

## Data Availability Statement

The raw data supporting the conclusions of this article will be made available by the authors, without undue reservation.

## Ethics Statement

The studies involving human participants were reviewed and approved by Medical Scientific Research Ethics Committee of Peking University Third Hospital. The patients/participants provided their written informed consent to participate in this study.

## Author Contributions

DF designed the study, interpreted the results, and revised the manuscript. QY designed the study, analyzed the data, and wrote the article. ZY, XL, LT, GZ, DT, and QY acquired the data. NL helped in data analysis, interpreted the results, and revised the manuscript. XZ helped in image assessment. All authors contributed to the article and approved the submitted version.

## Funding

This work was supported by National Natural Science Foundation of China (grant numbers 81901204, 81901298) and Beijing Municipal Science and Technology Commission (grant numbers D141100000114005).

## Conflict of Interest

The authors declare that the research was conducted in the absence of any commercial or financial relationships that could be construed as a potential conflict of interest.

## Publisher's Note

All claims expressed in this article are solely those of the authors and do not necessarily represent those of their affiliated organizations, or those of the publisher, the editors and the reviewers. Any product that may be evaluated in this article, or claim that may be made by its manufacturer, is not guaranteed or endorsed by the publisher.
