# Supplementary material for: CT-Visible Convexity Subarachnoid Hemorrhage Predicts Early Recurrence of Lobar Hemorrhage
Source: Front Neurol. 2022 Mar 23;13:843851. doi: 10.3389/fneur.2022.843851 (PMC8983869; doi:10.3389/fneur.2022.843851)
Supplement: Supplementary file 1 [file Data_Sheet_1.docx]

Table S1. Locations of the index and recurrent ICH.

| No. | location of the index ICH | Location of the recurrent ICH |
| --- | --- | --- |
| 1 | Left parietal lobe | Left frontoparietal lobe |
| 2 | Right lobes | Left temporal lobe |
| 3 | Left temporal lobe | Right temporoparietal lobe |
| 4 | Right parietal lobe | Left parietal lobe |
| 5 | Left occipital lobe | Left occipital lobe |
| 6 | Bilateral lobes | Right frontal lobe |
| 7 | Left frontal lobe | Right frontal lobe |
| 8 | Left temporal lobe | Left frontal lobe |
| 9 | Left parietooccipital lobe | Right temporal lobe |
| 10 | Left temporoparietal and right temporal lobe | Bilateral frontal and right parietal lobe |
| 11 | Left temporoparietal lobe | Left temporal parietal occipital lobe |
| 12 | Right lobes | left frontal lobe and right parietooccipital lobe |
| 13 | Right frontal lobe | Left frontoparietal lobe |
| 14 | Left temporal lobe | Right frontal parietal temporal lobe |
| 15 | Right parietal lobe | Right temporal lobe |
| 16 | Left frontal lobe | Right frontal lobe |
| 17 | Left temporal parietal occipital lobe | Lobes of brain |

Table S2. Baseline characteristics and comparison of probable CAA patients with and without cSAH

|  | Patients with cSAH | Patients without cSAH | P-value |
| --- | --- | --- | --- |
| No. of patients | n=25 | n=25 |  |
| Demographics |  |  |  |
| Age, Median [IQR] | 80 [71-82] | 73 [67-79] | 0.058 |
| Male, No. (%) | 15 (60.0) | 15 (60.0) | 1.000 |
| Clinical characteristics |  |  |  |
| Hypertension, No. (%) | 12 (48.0) | 14 (56.0) | 0.571 |
| Diabetes mellitus^a^, No. (%) | 2 (8.7) | 3 (12.0) | 1.000 |
| Hyperlipidemia^b^, No. (%) | 1 (4.8) | 5(20.0) | 0.276 |
| Previous symptomatic ICH, No. (%) | 15 (60.0) | 16(64.0) | 0.771 |
| Previous antiplatelet use^c^, No. (%) | 3 (12.5) | 5 (20.8) | 0.699 |
| Previous anticoagulant use^d^, No. (%) | 1(4.2) | 0(0) | 0.984 |
| NIHSS score^e^, Median [IQR] | 11 [4-23] | 5 [1-6] | 0.036 |
| Surgical treatment, No. (%) | 2 (8.0) | 2 (8.0) | 0.041 |
| Hospital length of stay^f^, Median [IQR] | 22 [13-38] | 17 [13-26] | 0.180 |
| ICH volume (mL), Median [IQR] | 29.8 [10.8- 52.1] | 10.0 [3.8-26.5] | 0.017 |
| Intraventricular hemorrhage presence, No. (%) | 12 (48.0) | 6 (24.0) | 0.077 |
| APOE ɛ2 (≥1 copy) ^f^, n (%) | 8 (42.1) | 5 (27.8) | 0.362 |
| APOE ɛ4 (≥1 copy) ^f^, n (%) | 5 (26.3) | 5 (27.8) | 1.000 |

^a^ 2 patients with missing data.

^b^ 4 patients with missing data.

^c^ 2 patients with missing data.

^d^ 1 patients with missing data.

^e^ 4 patients with missing data.

^f^ 37 patients consented to APOE genotype testing.

Abbreviations: cSAH, convexity subarachnoid hemorrhage; ICH, intracerebral hemorrhage; IQR, interquartile range.

Table S3. Univariate regression analysis of factors for recurrent ICH within 2 weeks in probable CAA patients

| Variables | P-value | OR | 95% CI |
| --- | --- | --- | --- |
| Age | 0.870 | 1.008 | 0.916- 1.109 |
| Male | 0.353 | 0.444 | 0.080- 2.465 |
| Hypertension | 0.113 | 0.250 | 0.045- 1.386 |
| Diabetes mellitus^a^ | 0.833 | 1.286 | 0.124- 13.295 |
| Hyperlipidemia^b^ | 0.999 | - | - |
| Previous symptomatic ICH | 0.975 | 1.026 | 0.215- 4.886 |
| Previous antiplatelet use^c^ | 0.730 | 0.673 | 0.071- 6.380 |
| Previous anticoagulant use^d^ | 1.000 | - | - |
| NIHSS score^e^ | 0.779 | 1.011 | 0.936- 1.093 |
| ICH volume | 0.073 | 1.021 | 0.998- 1.045 |
| Presence of cSAH | 0.149 | 3.632 | 0.656- 20.115 |
| Adjacent cSAH | 0.102 | 3.718 | 0.771- 17.938 |
| Remote cSAH | 0.894 | 0.857 | 0.089- 8.268 |
| Intraventricular hemorrhage presence | 0.374 | 2.000 | 0.434- 9.210 |

^a^ 2 patients with missing data.

^b^ 4 patients with missing data.

^c^ 2 patients with missing data.

^d^ 1 patients with missing data.

^e^ 4 patients with missing data.

Abbreviations: cSAH, convexity subarachnoid hemorrhage; ICH, intracerebral hemorrhage.

Table S4. Univariate regression analysis of factors for recurrent ICH within 90 days in probable CAA patients^a^

| Variables | P-value | OR | 95% CI |
| --- | --- | --- | --- |
| Age | 0.517 | 1.028 | 0.945- 1.119 |
| Male | 0.865 | 1.122 | 0.297- 4.240 |
| Hypertension | 0.189 | 0.400 | 0.102- 1.572 |
| Diabetes mellitus^b^ | 0.849 | 0.800 | 0.080- 8.007 |
| Hyperlipidemia^c^ | 0.999 | - | - |
| Previous symptomatic ICH | 0.731 | 0.791 | 0.208- 3.004 |
| Previous antiplatelet use^d^ | 0.353 | 0.351 | 0.038- 3.195 |
| Previous anticoagulant use^e^ | 1.000 | - | - |
| NIHSS score^f^ | 0.378 | 1.030 | 0.965- 1.099 |
| ICH volume | 0.104 | 1.018 | 0.996- 1.039 |
| cSAH presence | 0.015 | 7.857 | 1.495- 41.302 |
| Adjacent cSAH | 0.004 | 9.000 | 1.991- 40.691 |
| Remote cSAH | 0.618 | 0.564 | 0.059- 5.372 |
| Intraventricular hemorrhage presence | 0.063 | 3.640 | 0.934- 14.179 |

^a^ 2 patients were excluded because of missing data on ICH recurrence within 90 days

^b^ 1 patients with missing data.

^c^ 3 patients with missing data.

^d^ 2 patients with missing data.

^e^ 1 patients with missing data.

^f^ 3 patients with missing data.

Abbreviations: cSAH, convexity subarachnoid hemorrhage; ICH, intracerebral hemorrhage.

Table S5. Multivariate regression analysis of predictors of ICH recurrence within 90 days in probable CAA patients

| Variables | P-value | OR | 95% CI | Variables | P-value | OR | 95% CI |
| --- | --- | --- | --- | --- | --- | --- | --- |
| Model 1A^a^ | | | | Model 1B^a^ | | | |
| IVH presence | 0.158 | 2.856 | 0.664- 12.282 | IVH presence | 0.201 | 2.664 | 0.593-11.979 |
| cSAH presence | 0.026 | 6.791 | 1.254- 36.777 | Adjacent cSAH | 0.012 | 9.343 | 1.627-53.647 |
|  |  |  |  | Remote cSAH | 0.593 | 2.046 | 0.148-28.374 |
| Model 2A^b^ | | | | Model 2B^b^ | | | |
| Age | 0.710 | 1.019 | 0.922-1.126 | Age | 0.661 | 1.023 | 0.924-1.132 |
| Previous symptomatic ICH | 0.761 | 0.764 | 0.135-4.324 | Previous symptomatic ICH | 0.911 | 0.904 | 0.153-5.324 |
| Previous antiplatelet use | 0.452 | 0.366 | 0.027-5.030 | Previous antiplatelet use | 0.973 | 1.051 | 0.061-17.958 |
| Previous anticoagulants use | 1.000 | - | - | Previous anticoagulant use | 0.999 | - | - |
| cSAH presence | 0.033 | 6.553 | 1.165-36.870 | Adjacent cSAH | 0.013 | 9.814 | 1.606-59.959 |
|  |  |  |  | Remote cSAH | 0.999 | - | - |
| Model 3A^c^ | | | | Model 3B^c^ | | | |
| cSAH presence | 0.014 | 8.077 | 1.523- 42.834 | Previous anticoagulant use | 1.000 | - | - |
|  |  |  |  | Adjacent cSAH | 0.004 | 12.500 | 2.258-69.192 |

^a^ Variables with a P value < 0.1 in the univariate logistic analysis were included. 2 patients were excluded because of missing follow-up data on recurrence.

^b^ Prespecified plausible predictors of recurrent ICH were included. 4 patients were excluded because of missing data.

^c^ Prespecified plausible predictors as well as variables with a P value < 0.1 in univariable regression were included using backward logistic regression. Age, previous ICH, previous antiplatelet use and anticoagulant use, cSAH and IVH were entered, among which 5 variables, including Age, previous ICH, previous antiplatelet and anticoagulants use, and IVH were eliminated in the final model as backward logistic regression in model 3A. Age, previous ICH, previous antiplatelet use and anticoagulant use, IVH, adjacent and remote cSAH were entered, among which 5 variables, including age, previous ICH, previous antiplatelet use, IVH, and remote cSAH. were eliminated in the final model as backward logistic regression in model 3B. 4 patients were excluded because of missing data.

Abbreviations: cSAH, convexity subarachnoid hemorrhage; ICH, intracerebral hemorrhage; IVH, intraventricular hemorrhage.

Table S6**.** Baseline characteristics and comparison in patients with available MRI^a^

|  | Patients with cSAH | Patients without cSAH | P-value |
| --- | --- | --- | --- |
| No. of patients | n=32 | n=49 |  |
| Demographics |  |  |  |
| Age, Median [IQR] | 79 [73-82] | 71 [65-78] | 0.001 |
| Male, No. (%) | 19 (59.4) | 29 (59.2) | 0.986 |
| Clinical characteristics |  |  |  |
| Hypertension, No. (%) | 13 (40.6) | 25 (51.0) | 0.359 |
| Diabetes mellitus, No. (%) | 7 (21.9) | 10 (20.4) | 0.874 |
| Hyperlipidemia^b^, No. (%) | 1 (3.3) | 7(15.2) | 0.205 |
| Previous symptomatic ICH, No. (%) | 3 (9.4) | 10 (20.4) | 0.186 |
| Previous antiplatelet use^c^, No. (%) | 5 (16.1) | 13 (28.3) | 0.217 |
| Previous anticoagulant use^d^, No. (%) | 0 (0) | 0 (0) | - |
| NIHSS score^e^, Median [IQR] | 2.0 [1-6.5] | 2.0 [0-5.0] | 0.551 |
| Surgical treatment, No. (%) | 0 (0) | 2 (4.1) | 0.671 |
| Hospital length of stay, Median [IQR] | 24.0 [14.5-35.5] | 19.0 [14.0-25.0] | 0.087 |
| ICH volume (mL), Median [IQR] | 22.5 [8.2- 36.0] | 11.6 [4.1-22.0] | 0.008 |
| Intraventricular hemorrhage presence, No. (%) | 6 (18.8) | 8 (16.3) | 0.778 |
| MRI marker |  |  |  |
| Presence of cSS, No. (%) | 19 (59.4) | 16 (32.7) | 0.018 |
| Presence of CMB, No. (%) | 12 (37.5) | 35 (71.4) | 0.002 |
| CMB count, median [IQR], | 0 [0-2] | 2[0-9] | 0.005 |
| Fazekas WMH score^f^, median [IQR] | 3 [2-4] | 2 [2-4] | 0.711 |
| High degree of CSO-EPVS^g^, No. (%) | 0 (0) | 5 (10.9) | 0.154 |
| High degree of BG-EPVS^g^, No. (%) | 0 (0) | 4 (8.7) | 0.245 |
| APOE ɛ2 (≥1 copy)^h^, n (%) | 4 (26.7) | 7 (22.6) | 1.000 |
| APOE ɛ4 (≥1 copy) ^h^, n (%) | 5 (33.3) | 6 (19.4) | 0.501 |

^a^ 81 patients with available T2*GRE/SWI images.

^b^ 5 patients with missing data.

^c^ 4 patients with missing data.

^d^ 1 patients with missing data.

^e^ 1 patients with missing data.

^f^ 2 patients without available FLAIR images.

^g^ 4 patients without available T2-weighted images.

^h^ 46 patients consented to APOE genotype testing.

Abbreviations: cSAH, convexity subarachnoid hemorrhage; ICH, intracerebral hemorrhage; cSS, cortical superficial siderosis; CMB, cerebral microbleed; WMH, white matter hyperintensities; CSO, centrum semiovale; BG, basal ganglia; EPVS, enlarged perivascular spaces; IQR, interquartile range.

Table S7. Univariate regression analysis of factors for recurrent ICH within 2 weeks in patients with available MRI^a^

| Variables | P-value | OR | 95% CI |
| --- | --- | --- | --- |
| Age | 0.882 | 1.008 | 0.908- 1.120 |
| Male | 0.350 | 0.344 | 0.037- 3.223 |
| Hypertension | 0.243 | 0.264 | 0.028- 2.468 |
| Diabetes mellitus | 0.955 | 0.938 | 0.098- 8.981 |
| Hyperlipidemia^b^ | 0.999 | - | - |
| Previous symptomatic ICH | 0.157 | 3.939 | 0.589- 26.335 |
| Previous antiplatelet use^c^ | 0.375 | 2.333 | 0.358- 15.192 |
| Previous anticoagulant use^d^ | - | - | - |
| NIHSS score^e^ | 0.302 | 1.059 | 0.950-1.179 |
| ICH volume | 0.529 | 0.979 | 0.915-1.046 |
| Presence of cSAH | 0.346 | 2.431 | 0.383- 15.431 |
| Adjacent cSAH | 0.262 | 2.885 | 0.453- 18.362 |
| Remote cSAH | 0.999 | - | - |
| Intraventricular hemorrhage presence | 0.999 | - | - |
| MRI marker |  |  |  |
| Presence of cSS, | 0.124 | 5.806 | 0.619- 54.463 |
| Presence of CMB | 0.926 | 1.091 | 0.172- 6.912 |
| CMB count, median | 0.697 | 1.015 | 0.843- 1.091 |
| Fazekas WMH score^f^ | 0.366 | 1.336 | 0.713- 2.504 |
| High degree of CSO-EPVS^g^ | 0.240 | 4.250 | 0.381-47.423 |
| High degree of BG-EPVS^g^ | 0.999 | - | - |

^a^ 81 patients with available T2*GRE/SWI images.

^b^ 5 patients with missing data.

^c^ 4 patients with missing data.

^d^ 1 patients with missing data.

^e^ 1 patients with missing data.

^f^ 2 patients without available FLAIR images.

^g^ 4 patients without available T2-weighted images.

Abbreviations: cSAH, convexity subarachnoid hemorrhage; ICH, intracerebral hemorrhage; cSS, cortical superficial siderosis; CMB, cerebral microbleed; WMH, white matter hyperintensities; CSO, centrum semiovale; BG, basal ganglia; EPVS, enlarged perivascular spaces.

Table S8. Univariate regression analysis of factors for recurrent ICH within 90 days in patients with available MRI ^a^

| Variables | P-value | OR | 95% CI |
| --- | --- | --- | --- |
| Age | 0.163 | 1.071 | 0.973- 1.180 |
| Male | 0.596 | 0.672 | 0.155- 2.915 |
| Hypertension | 0.442 | 0.563 | 0.130- 2.435 |
| Diabetes mellitus | 0.412 | 0.406 | 0.047- 3.498 |
| Hyperlipidemia^b^ | 0.999 | - | - |
| Previous symptomatic ICH | 0.651 | 1.481 | 0.271- 8.095 |
| Previous antiplatelet use^c^ | 0.981 | 1.020 | 0.190- 5.472 |
| Previous anticoagulant use^d^ | - | - | - |
| NIHSS score^e^ | 0.195 | 1.059 | 0.971- 1.156 |
| ICH volume | 0.940 | 0.998 | 0.957- 1.042 |
| Presence of cSAH | 0.084 | 3.667 | 0.841- 15.986 |
| Adjacent cSAH | 0.047 | 4.476 | 1.021- 19.630 |
| Remote cSAH | 0.999 | - | - |
| Intraventricular hemorrhage presence | 0.175 | 2.900 | 0.622-13.525 |
| MRI marker |  |  |  |
| Presence of cSS | 0.047 | 5.315 | 1.026- 27.532 |
| Presence of CMB | 0.852 | 0.875 | 0.216- 3.551 |
| CMB count, median | 0.928 | 1.003 | 0.942- 1.067 |
| Fazekas WMH score^f^, | 0.884 | 1.038 | 0.626- 1.723 |
| High degree of CSO-EPVS^g^ | 0.594 | 1.875 | 0.186- 18.930 |
| High degree of BG-EPVS^g^ | 0.999 | - | - |

^a^ 4 patients were excluded from 81 patients with available MRI because of missing data on ICH recurrence within 90 days

^b^ 4 patients with missing data.

^c^ 4 patients with missing data.

^d^ 1 patients with missing data.

^e^ 1 patients with missing data.

^f^ 2 patients with missing data.

^g^ 4 patients with missing data.

Abbreviations: cSAH, convexity subarachnoid hemorrhage; ICH, intracerebral hemorrhage; cSS, cortical superficial siderosis; CMB, cerebral microbleed; WMH, white matter hyperintensities; CSO, centrum semiovale; BG, basal ganglia; EPVS, enlarged perivascular spaces.

Table S9. Multivariate regression analysis of predictors of ICH recurrence within 90 days in patients with available MRI ^a^

| Variables | P-value | OR | 95% CI | Variables | P-value | OR | 95% CI |
| --- | --- | --- | --- | --- | --- | --- | --- |
| Model A | | | | Model B | | | |
| Presence of cSAH | 0.202 | 2.702 | 0.587- 12.438 | Adjacent cSAH | 0.109 | 3.475 | 0.757- 15.946 |
| Presence of cSS | 0.092 | 4.256 | 0.789- 22.946 | Presence of cSS | 0.093 | 4.225 | 0.785- 22.751 |

^a^ Variables with a P value < 0.1 in the univariate logistic analysis were included. 4 patients were excluded from 81 patients with available MRI because of missing data on ICH recurrence within 90 days

Abbreviations: cSAH, convexity subarachnoid hemorrhage; cSS, cortical superficial siderosis.
